# Comparative study between efficacy of Excimer light with topical Tacrolimus 0.1% versus excimer light with topical Bimatoprost 0.01% in treatment of facial Vitiligo

**DOI:** 10.1007/s00403-024-03054-5

**Published:** 2024-06-08

**Authors:** Mohamed S. Zaky, Rabie B. Atallah, Nada Taha, Ali El Abasy, Mohamed L. Elsaie

**Affiliations:** 1 Department of Dermatology, Venereology and Andrology, Damietta Faculty of Medicine, Al-Azhar University, Damietta, Egypt; 2 Department of Dermatology, Damietta Dermatology and Leprosy Hospital, Damietta, Egypt; 3 Department of Dermatology, Medical Research and Clinical Studies Institute, National Research Centre, Giza, Egypt

**Keywords:** Vitiligo, Tacrolimus, Bimatoprost, Excimer

## Abstract

Loss and absence of melanocytes due to a number of factors is responsible for vitiligo; known to be the commonest disorder of pigmentation. The aim of the current work was to compare the efficacy and safety of excimer light with topical tacrolimus ointment 0.1% versus excimer light with topical bimatoprost gel 0.01% in treatment of facial vitiligo. The study was carried out on 48 patients presented with facial vitiligo. The patients were divided randomly using sealed envelope method into two groups (24 patients each). Group 1 were treated with excimer light plus topical tacrolimus ointment 0.1% and group 2 treated with excimer light plus topical bimatoprost gel 0.01%. Clinical improvement based on the quartile grading scale at the end of treatment did not show any statistically significant difference between groups. The majority of subjects in both groups experienced good to excellent improvement. Only 20.9% of patients in group 1 and 33.3% of subjects in group 2 achieved less than 50% repigmentation (*p* = 0.889). Our study demonstrated that 0.01% topical bimatoprost gel in combination with excimer light is considered safe and effective as treatment of nonsegmental facial vitiligo with comparable results to 0.1% tacrolimus.

## Introduction

Melanocytes are pigment cells available in both the epidermis and in hair follicles. Loss and absence of melanocytes due to a number of factors is responsible for vitiligo; known to be the commonest disorder of pigmentation. Vitiligo is commonly diagnosed before the age of thirty affecting roughly 1% of population (prevalence reported to be between 0.06% and 2.28%) with no sex predilection [1].

A number of genetic, environmental and behavioral interactions are among the contributing factors for vitiligo susceptibility and despite of the much advancement in disease understanding; yet the exact pathogenic mechanisms remains to be further elucidated. Similar to other autoimmune conditions; intrinsic and extrinsic cellular pathways play an important role in autodestruction of melanocytes by reactive CD8 + T cells [2].

Vitiligo management is challenging and choice of treatment modality depends on disease type and extent. Phototherapy is the first-line treatment for generalized vitiligo, while topical corticosteroids and topical calcineurin inhibitor are the first-line treatment for localized diseases with a high rate of repigmentation. Immunosuppressants, such as tacrolimus, have also been recommended; however, better results have been achieved by the introduction of UV light therapy to the treatment regimen. Prostaglandin analogues alone or in combination with phototherapy have shown positive results by assisting repigmentation [3].

One effective phototherapy device for the treatment of vitiligo is the 308 nm excimer laser (EL), which provides high precision and the ability to deliver a specific wavelength (308 nm) of radiation to target tissues over a short period. The EL has movable beam transmission capabilities that allow for selective light delivery to the specific lesion while maintaining healthy skin, thus reducing the risk of erythema in the surrounding depigmented area from overexposure, a common side effect in other phototherapy procedures [4].

Recent studies demonstrated an equipotent effect of tacrolimus to steroids in reaching 75% repigmentation and with extensively less side effects such as skin atrophy and telengeictasia. The effect of EL with tacrolimus ointment showed a higher reported synergestic effect to either treatment alone [5, 6].

Previous reports have suggested a successful outcome with topical bimatoprost; a synthetic prostaglandin analogue; as a treatment for facial or non facial vitiligo [7, 8]. Moreover; the efficacy and safety of 0.01% bimatoprost solution was comparable to 0.1% tacrolimus ointment in patients with facial vitiligo [9]. Bimatoprost combination with NB-UVB phototherapy was shown to be safe and effective for treating Thai patients with non-segmental vitiligo in non-face/neck areas of the body [10].

The aim of the current work was to compare the efficacy and safety of excimer light with topical tacrolimus ointment 0.1% versus excimer light with topical bimatoprost gel 0.01% in treatment of facial vitiligo.

## Patients and methods

This was a double armed single blinded randomized clinical trial study carried out after being approved by the local Ethics Committee of the Faculty of Medicine, Al-azhar University, Damietta, Egypt. The study was conducted over nine month’s duration, from March 2023 till Septmeber 2023. Ethical approval was obtained from the institutional review board of Damietta faculty of medicine (Al-Azhar University). All patients (or guardians if needed) were informed about the nature and the possible risks of the study and the details of the procedure and asked to provide written informed. Written informed consent was obtained from every patient at the recruitment.

The study was carried out on 48 patients presented with facial vitiligo. The patients were divided randomly using sealed envelope method into two groups (24 patients each). Group 1 were treated with excimer light plus topical tacrolimus ointment 0.1% and group 2 treated with excimer light plus topical bimatoprost gel 0.01%. Containers of applied tacrolimus and bimatoprost were identical and were different in color for each group. Each bottle was labeled with a container number, dosing instruction, and storage condition. Containers were provided to subjects and refilled on monthly basis.

Patients of any age and both sexes were included if they complained of stable facial vitiligo and have not received any form of vitiligo treatment during the last 3 months before recruitment. Stable vitiligo was determined if at least two items were identified: VIDA score ≤ 0; clinical feature of lesions with clear edges or signs of repigmentation; no Koebner phenomenon within 1 year; white lesion with sharply clear borders, smaller than or equal to the visual area under Wood’s light.

Exclusion criteria included pregnant or lactating females, Patients who had a history of photosensitivity, diseases exacerbated by sunlight, keloids or hypertrophic scars, were excluded, and those who showed sensitivity to the products were excluded from the study. Moreover Subjects with cognitive impairment, present psychiatric disorders were also excluded.

### Every patient was subjected to

#### Questioning about

1. Personal history including; name, age, sex, and residence.
2. Present history including; onset, course, and duration of vitiligo.
3. Past history of any chronic illness or associated autoimmune disease.
4. Family history of vitiligo, premature graying of hair, or other general diseases.
5. Drug history as previous phototherapy, oral or topical medications.

#### General and dermatological examination

- To exclude dermatological diseases other than vitiligo.
- All patients were subjected to carful dermatological examination in order to define the type and distribution of vitiligo, exclusion of any other skin problem as Koebners phenomenon.
- Wood’s lamp (Derma India, Chennai, India) and Dermoscopic (DermLite DL4,3 Gen, USA) examination were performed to confirm the diagnosis.

#### Preparation of bimatoprost gel

The first step was to dissolve 1% carbopol in distilled water (2 g is dissolved in 100 ml of distilled water under stirring conditions), the second step was to activate carbopol gel formation by using 0.01 gm or 0.03 gm of bimatoprost for every 100 ml of gel, and the third step was to incorporate the gel preparation into the base with 5 min of continuous stirring and stirring to obtain a homogeneous clear drug–gel solution. Bimatoprost gel was stored at room temperature in a dry place and not exposed to direct sunlight.

#### Treatment protocol

Patients were divided into two groups: The patients were randomly divided into 2 groups. Group 1 (*n* = 24) received excimer light (308-nm EL) with topical tacrolimus 0.1% ointment (308-nm EL + tacrolimus) and group 2 (*n* = 24) received excimer light with topical 0.01% bimatoprost gel (308-nm EL + bimatoprost).

In group 1 patients were instructed to apply tacrolimus ointment twice daily to the depigmented skin patch as a thin film sufficient to cover the affected area and then to rub it gently three to four times while in group 2; patients were instructed to use bimatoprost gel twice daily to the depigmented skin patch as a thin film sufficient to cover the affected area and then to rub it gently three to four times.

A 308-nanometer (nm) (Excilite µ™ (DEKA, Florence, Italy) was used, with a wavelength of 308 nm, a pulse width of 60 ns, light spot sizes of 10, 20 and 25 mm, and a light impulse energy of 6.5 mJ/cm2 with a repeat frequency of 200 Hz. The patients’ eyes were protected by special glasses. Treatment was provided twice a week on nonconsecutive days, and when a favorable outcome was observed, the treatment frequency was reduced to once a week for a total period of 3 months. The initial dose was 100 mJ/cm2, and subsequent doses were then increased by 20–30% every visit until slight erythema lasted 24–48 h. If tenderness or erythema occurred, the energy dose was maintained. If marked erythema with blisters developed, the treatment was withheld until the blisters disappeared. Both groups of patients were recommended to apply zinc oxide cream twice daily after and inbetween sessions.

#### Evaluation

All patient photographs were taken and documented by an expert before and upon completion of the treatment. Efficacy was blindly assessed by two independent expert dermatologists depending on the percentage of repigmentation. Repigmentation was evaluated by a quartile grading scale (QGS; grade 0, no repigmentation; grade 1, 1–25% minimal repigmentation; grade 2, 26–50% moderate repigmentation; grade 3, 51–75% good repigmentation; grade 4, 76–100% excellent repigmentation). The repigmentation was classified into four patterns, including perifollicular, marginal, diffuse and mixed patterns, which were identified using all study images. Adverse events and complications were recorded in every visit.

#### Follow-up and adverse events

Standard images were taken by a 16-megapixel digital camera (Canon Power Shot A3400 IS 16 MP digital camera; Tokyo; Japan) constantly at baseline, after 4weeks, 8weeks and after three months of treatment. Follow up including: Comparing the photographs before and after therapy; evolution of clinical response included degree of pigmentation and possible adverse effects including erythema, burning sensations and blister formation.

### Statistical analysis of the data

Data were fed to the computer and analyzed using IBM SPSS software package version 20.0. (Armonk, NY: IBM Corp) Qualitative data were described using number and percent. The Shapiro-Wilk test was used to verify the normality of distribution Quantitative data were described using range (minimum and maximum), mean, standard deviation, median and interquartile range (IQR). Significance of the obtained results was judged at the 5% level.

## Results

The demographic and clinical characteristics of the patients are summarized in Table 1. The baseline characteristics of the subjects in both groups were matched well. The average age of group 1 was 38.8 ± 11.4 years and im group 2 was 39.7 ± 10.9 years. Group 1 had 10 (41.7%) males and 14 (58.3%) females group 2 was comprised of 10 (41.7%) males and 14 (58.3%) females. Group 1 included 14 (58.3%) type III and 10 (41.7%) type IV Fitzpatrick skin phototype while group 2 included 15 (62.9%) III and 19 (67.9%) IV Fitzpatrick skin phototype (*p* = 0.768). All cases had a gradual onset, stationary course with a non significant median disease duration of 2 years ranging from 1 to 6 years for group 1 while ranging from 1 to 8 years in group 2 (*p* = 0.213). Tables 1 and 2.

**Table 1:** Comparison of demographic characteristics of the studied groups

	Group 1 *N* = 24(%)	Group 2 *N* = 24(%)	Test of significance
Age/ years			
Median (min-max)	13 (5–50)	13 (7–60)	Z = 0.041
Mean±SD	38,8±11,4	39,7±10,9	*P* = 0.967
Sex			
Male	10(41.7)	10(41.7)	FET, *P* = 1.0
Female	14(58.3)	14(58.3)	

Z: Mann Whitney U test, FET: Fischer exact test

**Table 2:** Comparison of disease characteristics between the studied groups

	Group 1 *N* = 24(%)	Group 2 *N* = 24(%)	Test of significance
Onset (gradual)	24(100)	24(100)	
course(last month) stationary	24(100)	24(100)	
Duration in years Median (min-max)	2(1–6)	1(1–8)	Z = 1.25 *P* = 0.213

Z: Mann Whitney U test

**Fig. 1:**
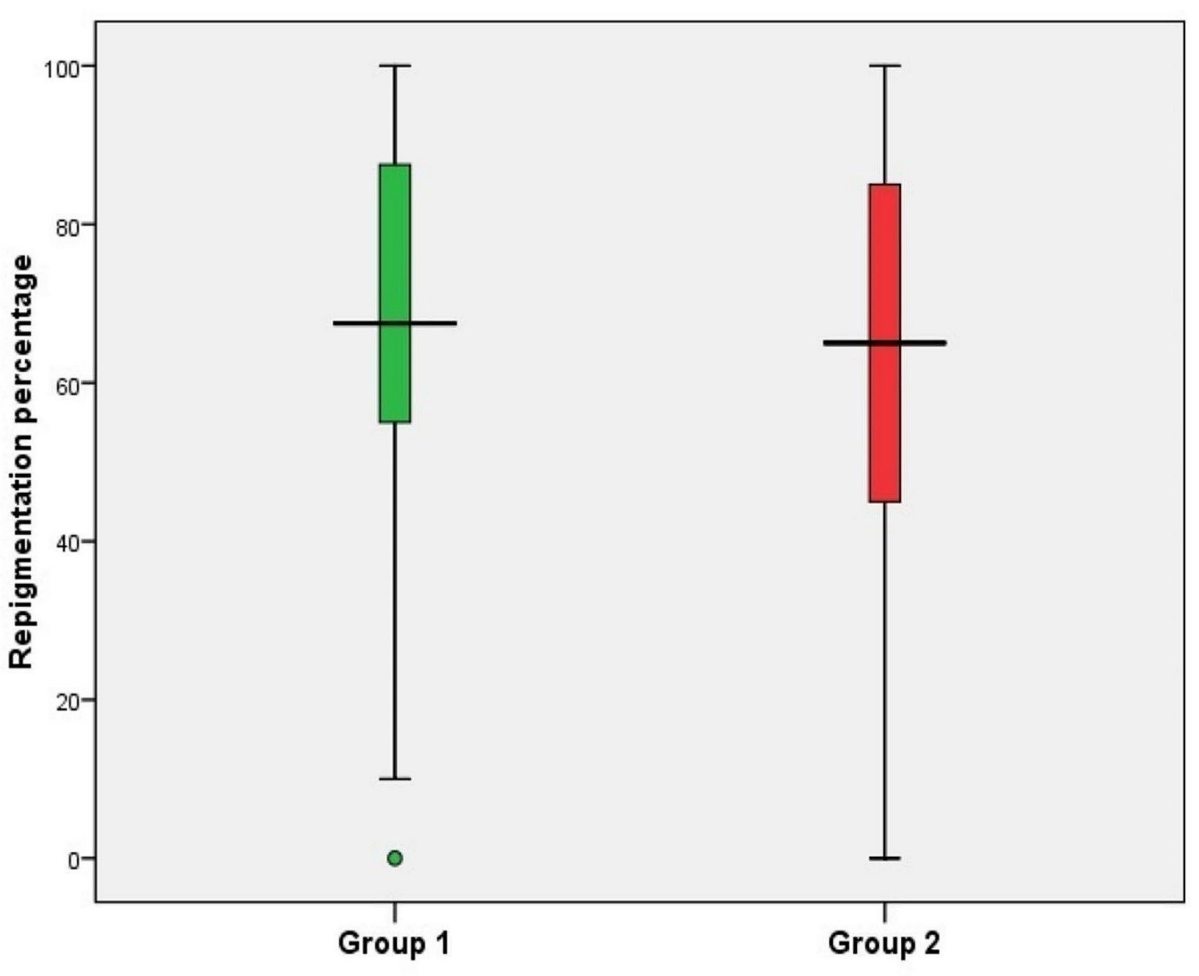
Box and Whisker plot showing re-pigmentation percentage among studied groups

**Fig. 2:**
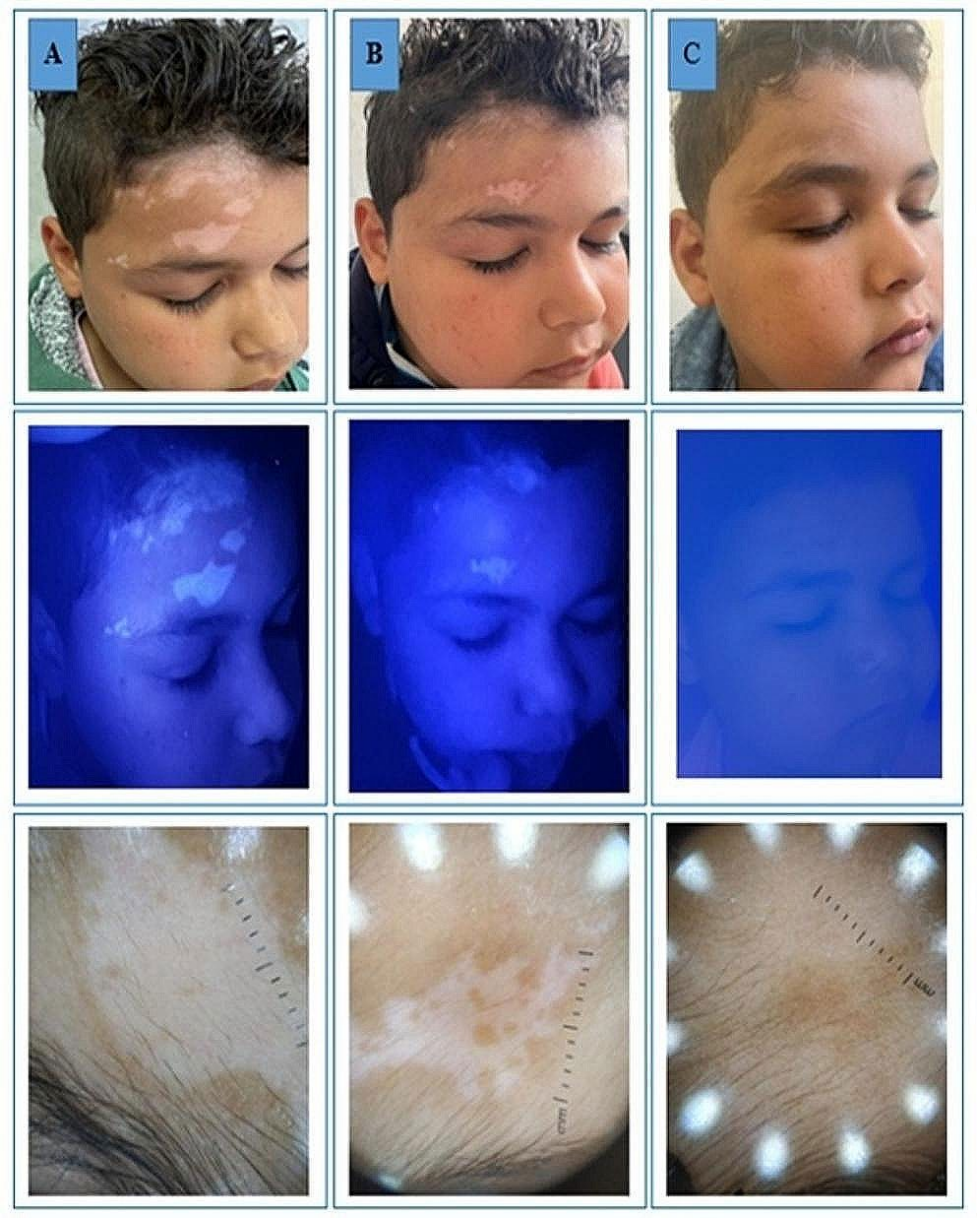
A 6-year old male presented with localized patch of facial vitiligo. **A**) At baseline, **B**) after 8 weeks and **C**) after 12 weeks of treatment with excimer light plus topical tacrolimus ointment 0.1%

**Fig. 3:**
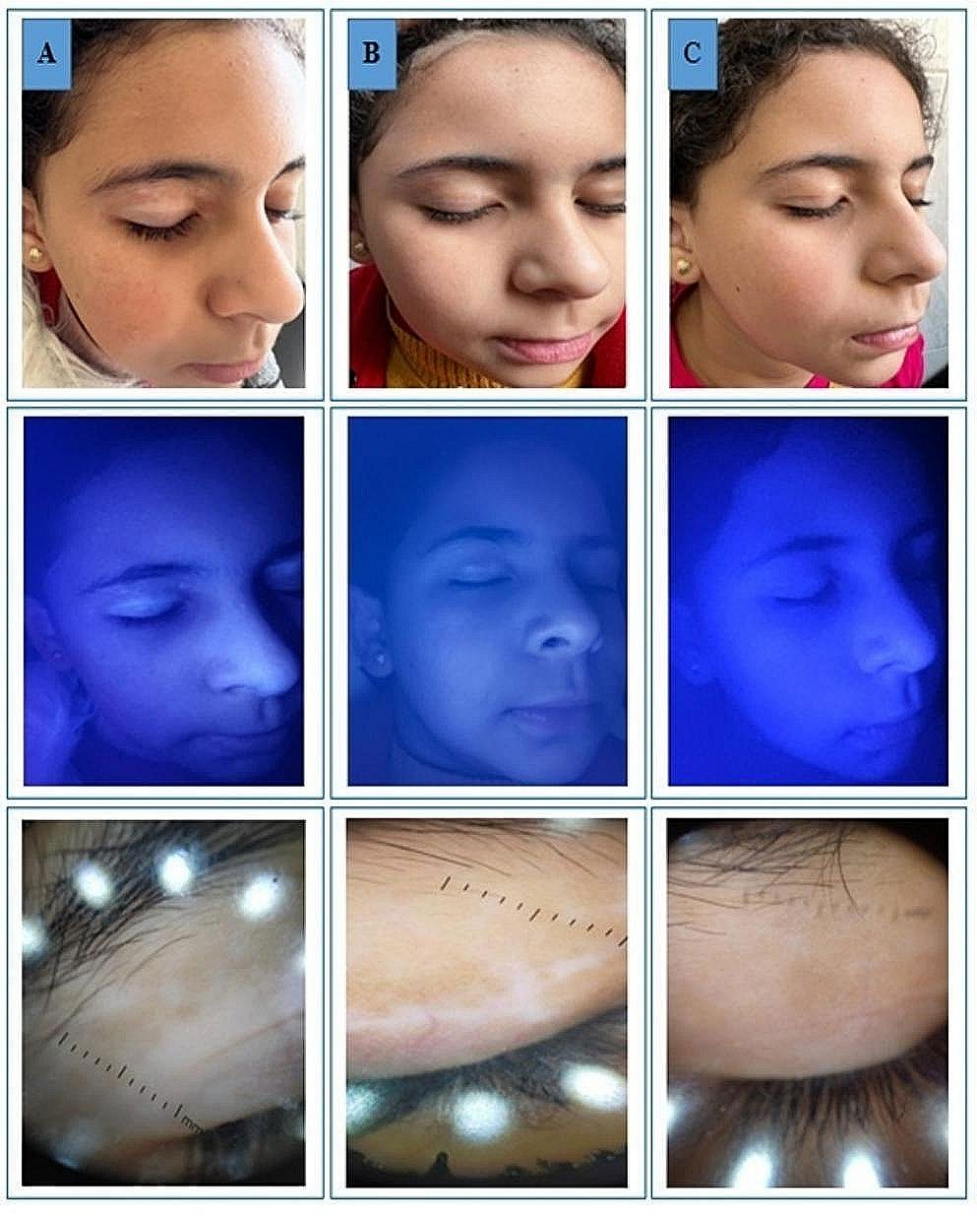
A 9-year old female presented with localized patch of facial vitiligo. **A**) At baseline, **B**) after 8 weeks and **C**) after 12 weeks of treatment with excimer light plus topical bimatoprost gel 0.01%

Clinical improvement based on the quartile grading scale at the end of treatment did not show any statistically significant difference between groups. The majority of subjects in both groups experienced good to excellent improvement. Only 20.9% of patients in group 1 and 33.3% of subjects in group 2 achieved less than 50% repigmentation (*p* = 0.889). The majority of patients reported good or excellent satisfaction with the results while only 8.3% of subjects in group 1 and 12.5% of subjects in group 2 demonstrated poor satisfaction (*p* = 0.875). As regard re-pigmentation pattern for group 1, 46% of patients had marginal re-pigmentation, 29% had diffuse re-pigmentation, 21% had perifollicular re-pigmentation and 4% had no re-pigmentation. In group 2; 38% of patients had marginal re-pigmentation, 25% had diffuse re-pigmentation, 33% had perifollicular re-pigmentation and 4% had no re-pigmentation. The patterns of repigmentation (marginal, diffuse and perifollicular) did not significantly differ among subjects of both groups (*p* = 0.809). Tables 3 and 4; Figs. 1, 2 and 3.

**Table 3:** Comparison of Quartile grading scale between the studied groups

Quartile grading scale	Group 1 *N* = 24(%)	Group 2 *N* = 24(%)	Test of significance
0	1(4.2)	1(4.2)	
1	1(4.2)	2(8.3)	
2	3(12.5)	5(20.8)	Mc = 1.14
3	10(41.7)	9(37.5)	*P* = 0.889
4	9(37.5)	7(29.2)	

MC Monte Carlo test

**Table 4:** Comparison of re-pigmentation pattern between the studied groups

Re-pigmentation pattern	Group 1 *N* = 24(%)	Group 2 *N* = 24(%)	Test of significance
No re-pigmentation	1(4.2)	1(4.2)	
Perifollicular	5(20.8)	8(33.3)	MC = 0.969
Marginal	11(45.8)	9(37.5)	*P* = 0.809
Diffuse	7(29.2)	6(25.0)	

MC: Monte Carlo test, Z:Mann Whitney U test, χ^22^ = Chi-Square test

The rates of repigmentation in both studied groups improved significantly more at week 12 when compared to weeks 4 and 8 (*p* < 0.001). There was however; no statistically significant difference between the rates of repigmentatiuon compared between both therapies at any time point in the study at weeks 4, 8 or 12 (*p* = 0.319; *p* = 0.205; *p* = 0.597). Table 5.

**Table 5:** Percent of improvement among studied groups (re-pigmentation percent)

	Group 1 *N* = 24	Group 2 *N* = 24	Test of significance
Week 4	20(0–50)	20(0–60)	Z = 0.997 *P* = 0.319
Week 8	50(0–80)	40(0–85)	Z = 1.27 *P* = 0.205
Week 12	67.5(0-100)	65(0-100)	Z = 0.528 *P* = 0.597
	P1 < 0.001* P2 < 0.001* P3 < 0.001*	P1 < 0.001* P2 < 0.001* P3 < 0.001*	

Percent of improvement is described as median (min-max), Z: Mann Whitney U test. p1: difference between week 4 & week 8, p2: difference between week 4 & 12, p3: difference between week 8& 12, used test Wilcoxon signed rank test

Mild side effects were reported and treatments were significantly tolerated in both treatment groups. A number of cases complained of irritation or desquamation while only 3 cases complained of vesicles in both groups that faded with dose adjustment with no other serious events recorded. Table 6.

**Table 6:** Side effects of treatment between studied groups

Side Effects	Group 1 *N* = 24(%)	Group 2 *N* = 24(%)	Test of significance
Blister	2(8.3)	1(4.2)	FET = 0.356 *P* = 1.0
Desquamation	4(16.7)	4(16.7)	FET = 0.0 *P* = 1.0
Irritation and itching	6(25.0)	4(16.7)	FET = 0.505 *P* = 0.724
Hypertrichosis	0	3(12.5)	FET = 3.2 *P* = 0.234
Hyperpigmentation	6(25.0)	4(16.7)	χ^2^ = 0.505 *P* = 0.477

FET Fischer exact test, χ^2^ = Chi-Square test

## Discussion

The following comparative study was conducted on 48 subjects reporting a mean age of 38.8 ± 11.4 and 39.7 ± 10.9 years in groups 1 and II respectively (*P* = 0.967). The age of onset in vitiligo and its subsequent progression is impelled by several inherited genes and can develop at any age irrespective of the type of skin, gender, race, or geographical location [2].

Periocular skin pigmentation was reported while treating ocular hypertension with prostaglandin F2 alpha analogs (PF2A). This observation led to an assumption of increased melanogenesis by prostaglandin analogues and provided a new role for its use in vitiligo [11].

Earlier reports concluded that analogues of PGF2α (latanoprost, bimatoprost, and travoprost) enhanced tyrosinase enzyme activity when combined with ultraviolet irradiation [12].

This randomized single-blind trial evaluated the the efficacy and safety of excimer light with topical tacrolimus ointment 0.1% versus excimer light with topical bimatoprost gel 0.01% in treatment of facial stable vitiligo. Both modalities produced a significant repigmentation with no statistically significant difference between the improvements compared between both of them at any time point in the study.

Different forms and concentrations of either bimatoprost or latanoprost proved to be effective in in vitiligo either alone or when compared to placebo [7, 8]. A proof-of-concept study performed by Grimes et al. [13]. to assess the efficacy of bimatoprost 0.03% ophthalmic solution in non-segmental vitiligo on non-facial areas compared to mometasone fuorate cream concluded that bimatoprost alone or with mometasone provided greater repigmentation than treatment with mometasone alone.

Repigmentation of nonfacial areas (e.g., extremities, neck, and trunk) has been observed in other studies assessing treatment of vitiligo with topical prostaglandins, including bimatoprost [8–10, 14]. Earlier reports by Parsad et al. [15]. used topical PGE2 for vitiligo, showed improvement in repigmentation while another study evaluating the safety and efficacy of topical prostaglandin E2 in treatment of vitiligo demonstrated that 50% of the lesions of up to 1 year duration of disease showed excellent to complete repigmentation [16].

Combination of topical bimatoprost and NB-UVB was reported more effective as compared to NB-UVB alone in the treatment of vitiligo vulgaris [17]. A randomized study using triple therapy of NB-UVB, fractional CO_2_ laser and topical bimatoprost 0.01% proved more efficacious to dual therapy of NB-UVB and fractional CO_2_ laser in the treatment of non-segmental vitiligo on non-facial areas [18]. Another study showed bimatoprost combination therapy to be safe and effective for treating non-segmental vitiligo in non-face/neck areas of the body [10].

Calcineurin inhibitors are immunomodulators and an off-label treatment for vitiligo functioning via inhibiting the lymphocyte and dendiric cells resident proinflammatory protein (calcineurin) that is responsible for the transcription of interleukin (IL)-2 and tumor necrosis factor-α (TNF-α). Such inflammatory cytokine inhibition results in the induction of melanocyte proliferation and activity [1].

A systemic metanalysis reviewed and reported the efficacy of tacrolimus as a monotherapy for the treatment of vitiligo [19]. Furthermore its efficacy was more superior when used in combination with either microneedling or dermabrasion to using it alone [20, 21]. Moreover; the combination of 308-nm excimer light and tacrolimus 0.1% ointment was superior to 308-nm excimer light alone in a number of studies [5, 6, 22].

Only one randomized, single-blinded and intra-individual controlled study compared the efficacy and safety of topical 0.01% bimatoprost compared with 0.1% tacrolimus for the treatment of facial vitiligo in 16 patients and demonstrated a significant efficacy of topical bimatoprost as a novel treatment option in facial vitiligo with comparable results to topical tacrolimus [9].

Only one unpublished report examined the efficacy of combining EL + topical bimatoporst versus EL alone in treating patients with vitiligo. Based on the results reported, EL combined with bimatoprost was significantly better in improving repigmentation when compared to EL monotherapy [23].

With reference to safety of the treatment, most patients treated by both techniques had no complication, however irritation, desquamation, hyperpigmentation and blister were observed in some patients especially treated with excimer plus tacrolimus with no significant difference between the two groups. Moreover; hypertrichosis was observed in 3 patients treated with excimer plus bimatoprost.

Our study had some limitations; the relatively small sample size. In addition, selection bias, as the patients were primarily recruited from patients who attended to our university clinic only. Another limitation was the short follow up of 3 months which could have not identified any late recurrences if any.

Our study demonstrated that 0.01% topical bimatoprost gel in combination with EL is considered safe and effective as treatment of nonsegmental facial vitiligo with comparable results to 0.1% tacrolimus. Further larger multi centre studies are required to establish its efficacy in different concentrations and formulations.

## Data Availability

The data that support the findings of this study are available from the corresponding author upon reasonable request.

## Abbreviations

EL: Excimer Light
IL-2: Interleukin 2
QGS: Quartile grading scale
NB-UVB: Naroow band ultraviolet B
PF2A: prostaglandin F2 alpha analogs
TBSE: Total Body Skin examination
TCI: Topical calcineurin inhibitors
TNF-α: Tumor Necrosis Factor‐alpha
UPR: Unfolded protein response
UTSG: Ultra-thin skin Grafting
UV: Ultraviolet
VASI: Vitiligo Area Scoring Index
VIDA: Vitiligo disease activity score

## Code Availability

All authors confirm all data and materials as well as software applications or custom code support their published claims and comply with field material.
